# Halide Mixing Inhibits Exciton Transport in Two-dimensional Perovskites Despite Phase Purity

**DOI:** 10.1021/acsenergylett.1c02403

**Published:** 2021-12-22

**Authors:** Michael Seitz, Marc Meléndez, Peyton York, Daniel A. Kurtz, Alvaro J. Magdaleno, Nerea Alcázar-Cano, Anuraj S. Kshirsagar, Mahesh K. Gangishetty, Rafael Delgado-Buscalioni, Daniel N. Congreve, Ferry Prins

**Affiliations:** † Condensed Matter Physics Center (IFIMAC), Autonomous University of Madrid, 28049 Madrid, Spain; ‡ Department of Condensed Matter Physics, Autonomous University of Madrid, 28049 Madrid, Spain; § Rowland Institute at Harvard University, Cambridge, Massachusetts 02142, United States; ∥ Department of Electrical Engineering, Stanford University, Stanford, California 94305, United States; ⊥ Department of Theoretical Condensed Matter Physics, Autonomous University of Madrid, 28049 Madrid, Spain; # Department of Chemistry, Mississippi State University, Mississippi State, Mississippi 39762, United States

## Abstract

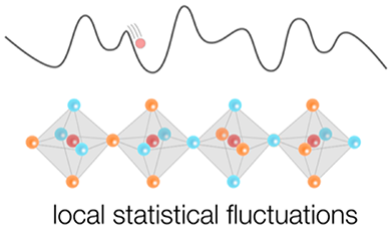

Halide mixing is one of the most powerful techniques to tune the optical bandgap of metal-halide perovskites. However, halide mixing has commonly been observed to result in phase segregation, which reduces excited-state transport and limits device performance. While the current emphasis lies on the development of strategies to prevent phase segregation, it remains unclear how halide mixing may affect excited-state transport even if phase purity is maintained. Here, we study exciton transport in phase pure mixed-halide 2D perovskites of (PEA)_2_Pb(I_1–*x*_Br_*x*_)_4_. Using transient photoluminescence microscopy, we show that, despite phase purity, halide mixing inhibits exciton transport. We find a significant reduction even for relatively low alloying concentrations. By performing Brownian dynamics simulations, we are able to reproduce our experimental results and attribute the decrease in diffusivity to the energetically disordered potential landscape that arises due to the intrinsic random distribution of alloying sites.

Metal-halide perovskites have become an important material platform for light-harvesting^1−4^ and light-emitting^5,6^ applications thanks to their numerous advantageous properties such as solution processability, high ambipolar charge-carrier mobilities,^7,8^ high defect tolerance,^9−11^ and variable optical properties.^12,13^ One key advantage of metal-halide perovskites is their widely tunable optical bandgap, which can be readily adjusted by introducing variations in the halide composition.^12−14^ Halide mixing has been widely employed in perovskite tandem solar cells to tune the bandgap and maximize the absorptive efficiency.^15,16^ In addition, halide mixing can be used in light-emitting devices (LEDs) to fine-tune the color of emission, a strategy that has been successfully employed for both bulk (3D) and layered (2D) perovskites.^17,18^

However, halide mixing has been associated with reduced bandgap stability as a result of ion migration.^19,20^ This instability is a result of the soft inorganic lattice of perovskites, which reduces activation energies for ion migration.^20^ In extreme cases, mixed-halide perovskites have been observed to undergo phase segregation, leading to extended regions of high and low energy sites. For example, in MAPb(I_1–*x*_Br_*x*_)_3_ 3D perovskites, it has been found that halide mixing yields a miscibility gap in the range of 30–90% of bromide concentration, causing the formation of iodide-rich and bromide-rich regions.^21^ In addition to this intrinsic miscibility gap, mixed-halide perovskites have been shown to suffer from light-induced phase segregation.^22−24^ Crucially, phase segregation can have a significant impact on carrier transport as carriers can get trapped in low energy regions.^19,24−27^ A number of strategies to avoid phase segregation in 3D perovskites are being developed, including mixed A-site cations,^19,28^ doping with tin (Sn), manganese (Mn), or potassium (K),^29,30^ or oxygen passivation.^31^ Interestingly, phase segregation appears to be absent in 2D perovskites.^12,32−36^ This may be attributed to the increased flexibility of the 2D lattice, which is more tolerant to local strain.

Importantly though, 2D mixed-halide alloys have been identified as pseudobinary alloys.^32,33^ In this case, even in the absence of phase segregation, statistical fluctuations in the distribution of alloy ions in the mixed-halide crystal can lead to *local* variations in the bandgap energy.^32,33^ Such site-to-site energetic disorder is commonly encountered in other pseudobinary alloy systems such as Al_*x*_Ga_1–*x*_As, ZnSe_*x*_Te_1–x_, or PbI_2(1-x)_Br_2*x*_.^32,33,37−39^ Indeed, Lanty et al. found that the optical properties of 2D mixed-halide perovskites (PEA)_2_Pb(I_1–*x*_Br_*x*_)_4_ are consistent with this mixed crystal picture, reproducing the change in position and width of the optical absorption spectrum for the different halide ratios by only accounting for the local statistical fluctuations.^33^ Therefore, despite having a phase-pure crystal lattice, the excited-state transport properties of 2D mixed-halide perovskites may still be affected by inhomogeneities in the energy landscape. For 3D perovskites, it has indeed been suggested that local inhomogeneities in the energy landscape of phase-pure mixed halide perovskites are at the origin of reduced charge carrier mobilities.^40^ To date, however, the vast majority of transport studies of mixed-halide perovskites have focused on the effects of phase segregation.^19,25,27^ As a result, the detailed role of local inhomogeneities in determining the excited-state transport of mixed-halide perovskites remains elusive.

In this study, we investigate the impact of halide mixing on excited-state transport in phase-pure single-crystalline 2D metal-halide perovskites of phenethylammonium lead (iodide/bromide) (PEA)_2_Pb(I_1–*x*_Br_*x*_)_4_ (*x* = 0–100%). We show that, despite the absence of phase segregation, halide mixing significantly inhibits the transport of the excitonic excited state. Specifically, we observe that for bromide concentrations of 25–95% the exciton transport drops by a factor of more than ten as compared to the pure phases. Using transient spectroscopy, we show that this regime of strongly decreased diffusivity coincides with a significant energetic disorder that is present in the material. Performing Brownian dynamics simulations and accounting for the statistical fluctuations in chemical composition, we are able to reproduce the spatial and spectral exciton dynamics observed in our experiments. Because of the excellent agreement between simulations and experiments, we conclude that the decrease of transport properties in mixed-halide 2D perovskites is a result of energetic disorder caused by the alloying sites. Importantly, our results show that even if phase segregation is eliminated, excited-state transport in metal-halide perovskites may still be significantly affected by halide mixing.

Single crystals of phenethylammonium lead (iodide/bromide) (PEA)_2_Pb(I_1–*x*_Br_*x*_)_4_ (*x* = 0–100%) 2D perovskites were synthesized from saturated precursor solutions (see methods).^41,42^ By varying the bromide fraction *x*, we obtain the typical optical bandgap tuning, as evidenced by the resulting photoluminescence (PL) spectra ranging from the deep-blue into the green (see Supporting Information (SI), Figure S1 and S2).^12,33^ Through mechanical exfoliation, we isolate single-crystalline flakes (10–100 μm in lateral size), which are transferred onto a microscopy slide for investigation with an oil immersion objective (see Figure 1c–e). Using thick flakes provides a form of self-passivation, as the surface of interest is protected by the thick crystal, providing a good diffusion barrier to oxygen and moisture for the duration of our measurements.^43^ To confirm phase purity of the single-crystalline flakes, we performed diffraction limited hyperspectral imaging, finding spatially homogeneous emission energies with no clear sign of phase segregation (see SI, Figure S3). In addition, we performed elemental mapping by using a scanning electron microscope equipped with an energy dispersive X-ray spectrometer (SEM-EDS). The EDS maps revealed spatial homogeneous distributions of lead, iodide, and bromide in PEA_2_Pb(Br_0.5_I_0.5_)_4_ perovskite (see SI, Figure S4).

**Figure 1:**
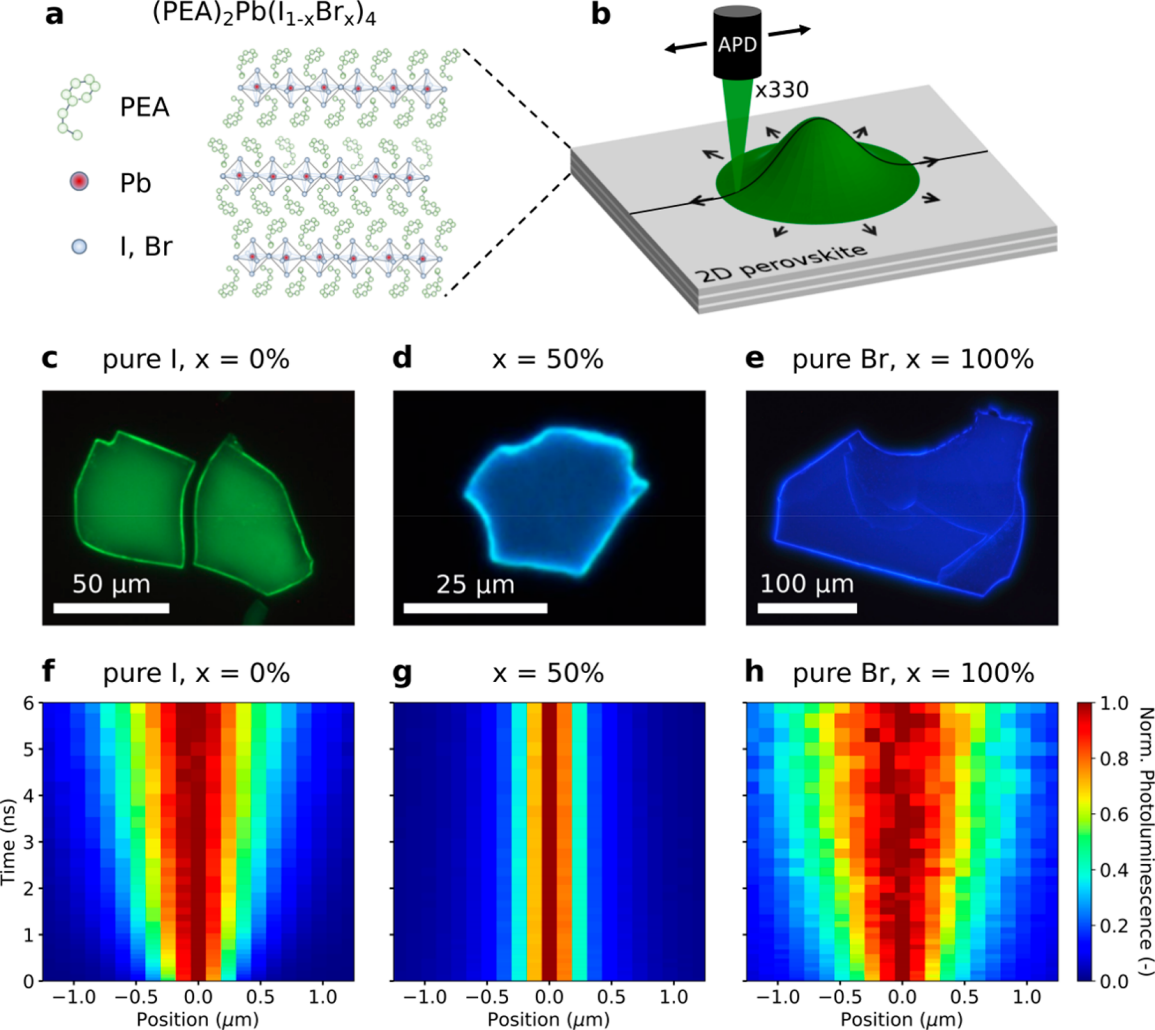
Diffusion imaging in 2D mixed-halide perovskites. (a) 2D perovskite crystal structure of (PEA)_2_Pb(I_1–*x*_Br_*x*_)_4_. (b) Transient photoluminescence microscopy setup displaying the emission spot of a narrow exciton population which broadens over time as excitons diffuse outward. The broadening is captured by magnifying the image of the emission 330 times and projecting it onto a scanning avalanche photodiode (APD) to track the evolution of excitons in space and time. (c–e) Fluorescence micrographs of 2D perovskite single-crystalline flakes with different halide ratios: *x* = 0, 50, or 100%. (f–h) Evolution of the emission cross-section *I*(*x*,*t*) for 2D perovskite single crystals with different halide ratios: *x* = 0, 50, or 100%. *I*(*x*,*t*) was normalized at each point in time to highlight the broadening of the exciton distribution.

In 2D metal-halide perovskites, the optical properties are dominated by excitonic excited states due to strong quantum and dielectric confinement effects.^44^ To follow the spatiotemporal evolution of photogenerated excitons, we use transient photoluminescence microscopy (TPLM), which allows the extraction of the excitonic transport properties along the 2D inorganic plane as described previously.^43,45−47^ In short, we create a narrow exciton population with a pulsed and near-diffraction limited laser diode (λ_ex_ = 405 nm) and an oil immersion objective (NA = 1.3). As excitons start diffusing, the exciton population broadens with time. Using a scanning avalanche photodiode, we follow the broadening of the exciton population by tracking its PL emission *I*(*x*,*t*) (see Figure 1a,b), which is proportional to the exciton density at low laser fluences. For isotropic materials such as 2D perovskites, it is sufficient to scan a 1D slice of the exciton population.^46,48^ The result of such a scan is presented in Figure 1f for (PEA)_2_PbI_4_ (*x* = 0%), where the broadening of the emission spot is highlighted by normalizing the emission cross-section *I*(*x*,*t*) at each point in time. At *t* = 0, we observe a near-diffraction limited emission spot, while at later times the emission is broadened due to the outward diffusion of excitons. The rate at which the emission broadens can be quantified by using the mean-square-displacement (MSD) of the emission cross-section and can be used to determine the diffusivity *D*, which describes the speed at which excitons travel through the single-crystal (see SI).^43,45,46^

For pure iodide (PEA)_2_PbI_4_ (*x* = 0%) and pure bromide (PEA)_2_PbBr_4_ (*x* = 100%), we find a high diffusivity with the PL cross-section quickly broadening as time goes by, indicating fast exciton diffusion (see Figure 1f,h). Specifically, we find a comparable diffusivity for pure iodide (*x* = 0%, *D* = 0.204 cm^2^ s^–1^) and pure bromide (*x* = 100%, *D* = 0.222 cm^2^ s^–1^). The diffusivity for pure iodide is consistent with our earlier studies, where we synthesized single crystals with the same synthetic procedure.^43,49,50^ However, when mixing equal amounts of iodide and bromide (*x* = 50%), we observe no broadening of the exciton distribution at all, as shown in Figure 1g, meaning that exciton diffusion is below our detection limit of around 20–40 nm.

To investigate the impact of halide mixing in greater detail, we synthesize perovskite single crystals with a wider range of different bromide contents (*x* = 0, 5, 10, 25, 50, 75, 90, 95, and 100%). The resulting diffusion maps and MSD plots are shown in SI, Figure S5–8, and the extracted diffusivities *D*(*x*) and resulting diffusion length *L*_*D*_(*x*) are shown in Figure 2. We observe that the diffusivity *D*(*x*) is highly affected by halide mixing even at low levels such as 5 and 95%. In addition, mixing has a more severe impact on the bromide-rich side, where the observed diffusivity rapidly drops below our detection limit already for *x* = 90%, while on the iodide-rich side, we still observe a measurable diffusivity for 25%. We would like to note that for the laser fluences used in this study, we also exclude light-induced phase segregation as the origin of energetic disorder and reduced diffusivity, as power-dependent TPLM measurements yield results that are independent of the laser fluence (see SI, Figure S12). This is consistent with recent reports by Kamat and co-workers, who showed that PEA-based 2D perovskites are resilient to light-induced phase segregation.^35,36^

**Figure 2:**
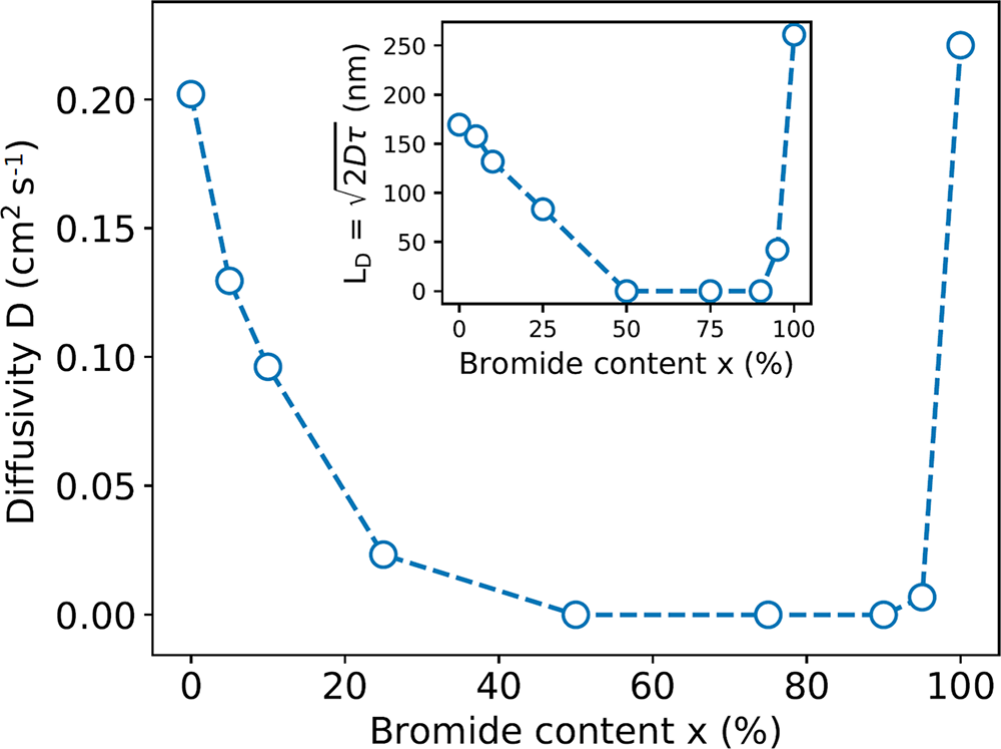
Diffusivity *D*(*x*) as a function of bromide content of various 2D metal-halide perovskites (PEA)_2_Pb_(_I_1–*x*_Br_*x*_)_4_ with *x* = 0, 5, 10, 25, 50, 75, 90, 95, and 100%. The inset shows the diffusion length , where τ(*x*) is the 1/*e* photoluminescence lifetime (see SI, Figure S9).

As reported in literature, significant broadening of the optical spectra is present for increased mixing in (PEA)_2_Pb(I_1–*x*_Br_*x*_)_4_ (see also SI, Figure S1).^30,32,33,51^ Lanty et al. showed that the spectral broadening in (PEA)_2_Pb(I_1–*x*_Br_*x*_)_4_ can be attributed to the presence of energetic disorder due to the local statistical fluctuation in chemical composition, resulting in a higher bandgap energy in bromide-rich locations and a lower energy in iodide-rich regions, consistent with other pseudobinary alloy systems.^32,33,37−39^ To analyze the possible correlation between a decrease in diffusivity and the presence of energetic disorder, we performed transient photoluminescence spectroscopy measurements using a streak camera. Figure 3a presents normalized streak camera images for the *x* = 0, 50, and 100% mixed-halide perovskites, showing how the PL spectra evolve as a function of time. The dashed lines highlight the shift of the maxima, while the solid lines correspond to the evolution of the median emission energy. In Figure 3b, we show the median emission energy for all halide mixtures. We observe the largest red-shifts for the mixtures which show the most dramatic decrease in diffusivity, consistent with a gradual energetically downhill transport of excitons in a disordered energy landscape. These results suggest that energetic disorder is indeed the origin of the observed trend in diffusivity. The correlation between the spatial and energetic dynamics is further emphasized by the observation of the same asymmetry in the full width at half-maximum (fwhm), the energy shift, as well as the diffusivity, all of which show a bigger impact of halide mixing on the bromide-rich side (see Figure 3c).

**Figure 3:**
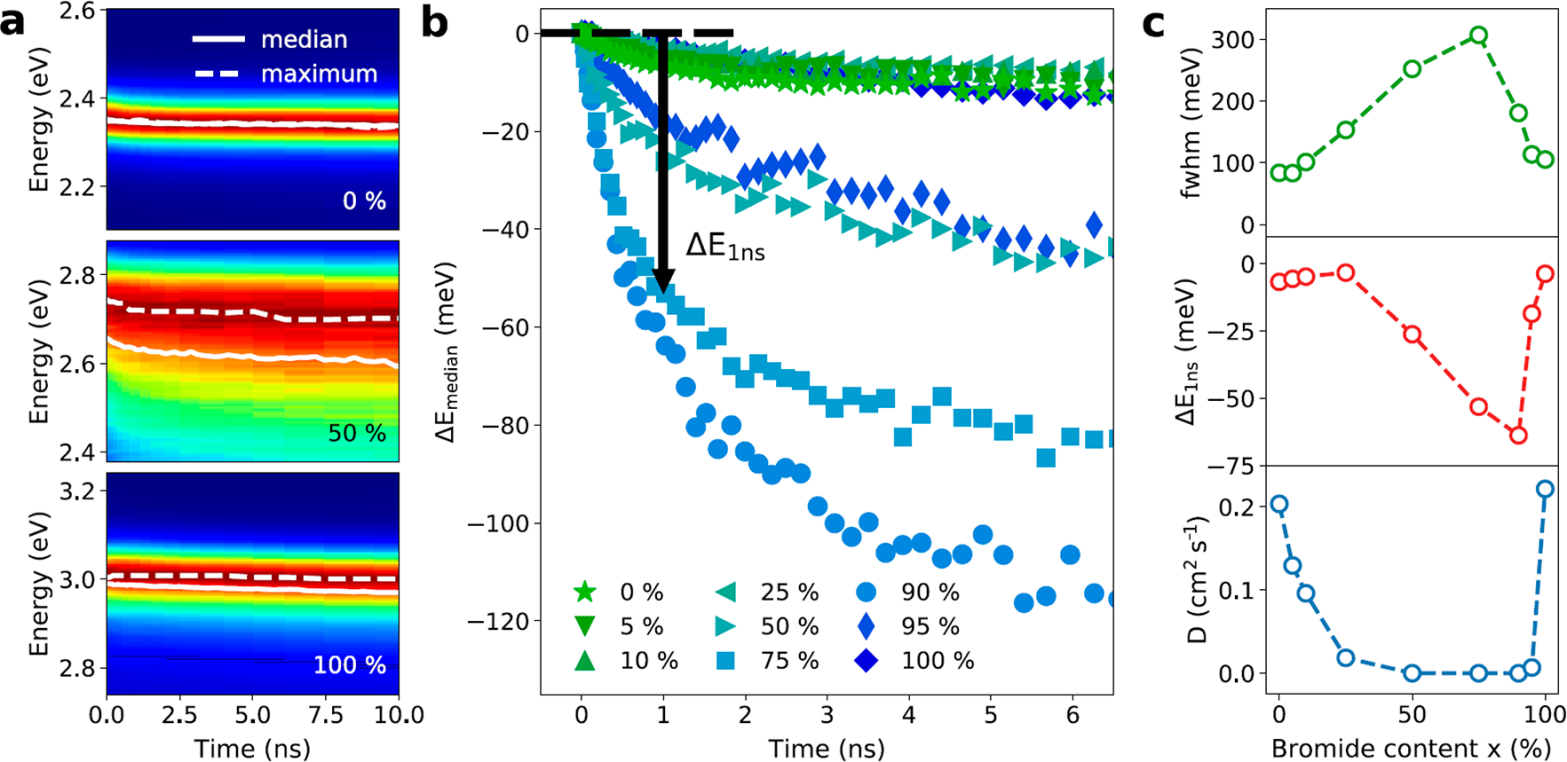
Spectrally resolved transient photoluminescence of (PEA)_2_Pb(I_1–*x*_Br_*x*_)_4_. (a) Streak camera images for *x* = 0, 50, and 100% show the evolution of the spectra over 10 ns. Spectra were normalized at each point in time. The dotted line tracks the evolution of the maximum, while the solid line shows the evolution of the median emission energy. (b) Evolution of the median emission energy for various halide mixtures with *x* = 0, 5, 10, 25, 50, 75, 90, 95, and 100%. (c) Full width at half-maximum (fwhm) of PL emission at *t* = 0 (top panel), median energy shift after 1 ns Δ*E*_1ns_ (center panel), and diffusivity *D* (bottom panel, same as Figure 2) as a function for bromide content x.

To obtain deeper insight into the relation between exciton transport and energetic disorder due to local statistical fluctuations in the halide composition, we performed Brownian dynamics simulations. The potential landscapes *V*(***r***) of the different halide mixtures are generated by randomly filling halide sites with iodide and bromide ions with a probability of (1 – *x*) and *x* (see SI). The probability density of a 2D exciton can be approximated with |Ψ(*R*)|^2^ ∝ *e*^–2*R*^2^/*a*_B_^2^^, where *R* is the distance from the center and *a*_B_ is the exciton Bohr radius (see SI).^52^ Convolving the iodide and bromide sites with the exciton’s probability density |Ψ(*R*)|^2^, we calculate the local bromide content *x*′(***r***) observed by an exciton at position ***r*** in the crystal. Note that *x*′(***r***) represents the *local* bromide content observed by the exciton at position ***r*** in the crystal, while *x* represents the *average* bromide content of the *whole* crystal. For 2D mixed-halide perovskites the bandgap has been shown to increase linearly with halide content.^33,53^ As a result, the local energy bandgap observed by an exciton at position ***r*** can be calculated as *E*_g_(*x*′(***r***)) = (1 – *x*′(***r***)) · *E*_g_^I^ + *x*′(***r***) · *E*_g_^Br^, where *E*_g_^I^ and *E*_g_^Br^ are the bandgaps of the pure iodide (*x* = 0%) and pure bromide (*x* = 100%) perovskites. The resulting potential landscapes *V*(***r***) (≡ *E*_g_(*x*′(***r***)) – min[*E*_g_(*x*′(***r***))]) for different bromide contents *x* are shown in SI, Figure S15, and Figure 4a and are used for the Brownian dynamics simulations (see SI). Note that we use *V*(***r***) for a better visualization and comparison of the different compositions *x*, instead of *E*_g_(***r***). For the Brownian dynamics simulations, we used Bohr radii that were previously reported for pure iodide (*a*_B_^I^ ≡ *a*_B_(*x* = 0%) = 1.15 nm) and pure bromide (*a*_B_^Br^ ≡ *a*_B_(*x* = 100%) = 0.7 nm) and linear interpolation for mixed components: *a*_B_(*x*) = (1 – *x*) · *a*_B_^I^ + *x* · *a*_B_^Br^.^14^ In addition, we approximate the diffusion coefficient *D*_0_ of mixed-halide perovskites, which describes the intrinsic diffusivity in the absence of any energetic disorder through linear interpolation between the two pure components: *D*_0_(*x*) = (1 – *x*) · *D*_0_^I^ + *x* · *D*_0_^Br^, with *D*_0_^I^ = *D*(*x* = 0%) = 0.204 cm^2^ s^–1^ and *D*_0_^Br^ = *D*(*x* = 100%) = 0.222 cm^2^ s^–1^ (cf. Figure 2).

**Figure 4:**
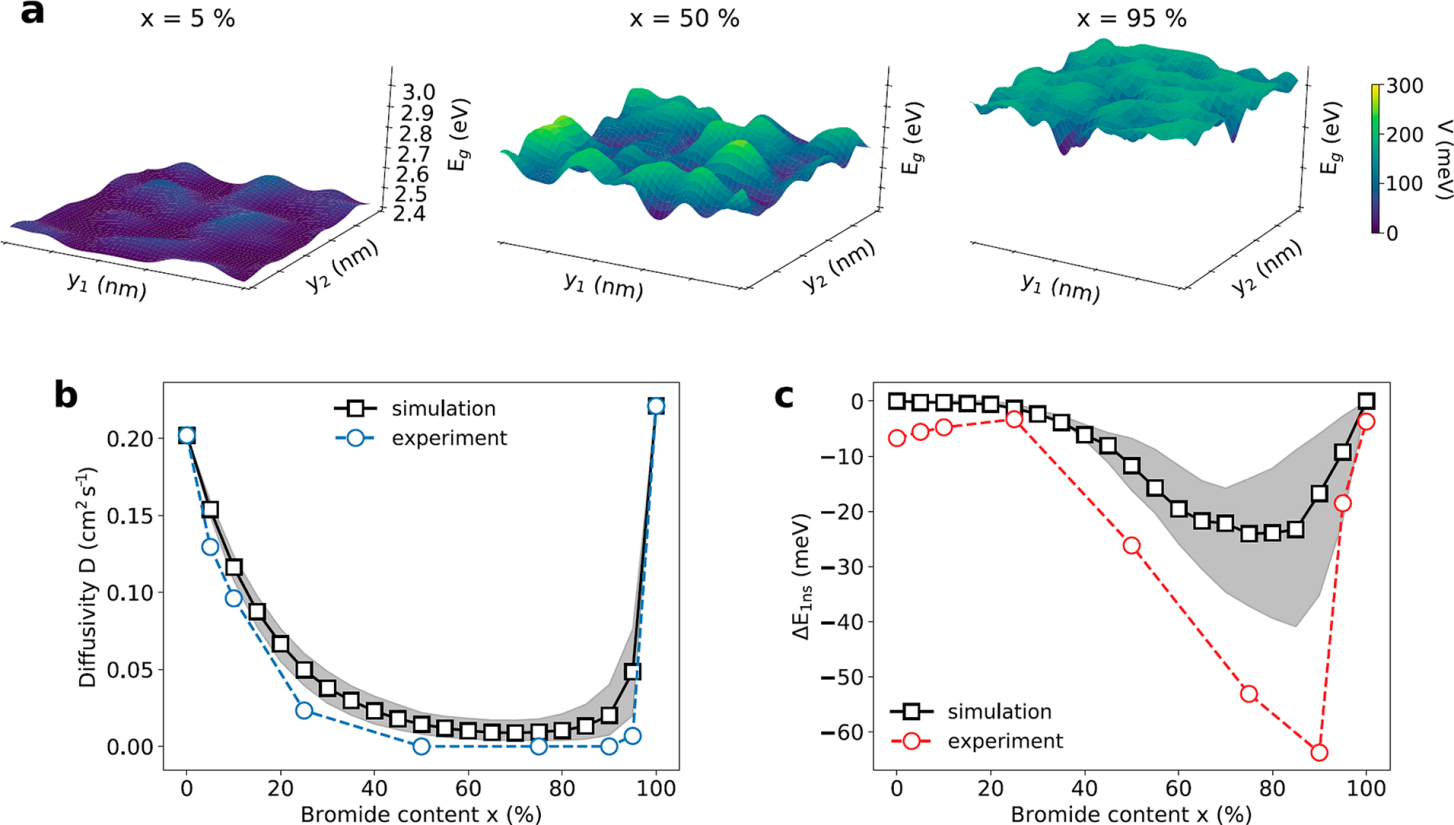
Experiments vs Brownian dynamics simulations. (a) Potential landscape *V*(*y*_1_, *y*_2_) for *x* = 5, 50, and 95% on a 10 × 10 nm^2^ area. (b) Diffusivity *D*(*x*) as a function of bromide content *x* comparing experiments (open circles) and Brownian dynamics simulations (open squares) with an exciton Bohr radius of *a*_B_(*x*) = (1 – *x*) · *a*_B_^I^ + *x* · *a*_B_^Br^ (*a*_B_^I^ = 1.15 nm, *a*_B_^Br^ = 0.7 nm). Shaded area shows simulation results with *a*_B_^I^ = 1.15 ± 0.1 nm and *a*_B_^Br^ = 0.7 ± 0.1 nm. (c) Change in median energy after 1 ns of photoexcitation Δ*E*_1ns_ (*x*) as a function of bromide content *x*. Simulated values were obtained from SI, Figure S19. We used a moving average () for the simulated data points in c.

The results of the Brownian dynamics simulations are shown in Figure 4b,c. We find excellent agreement with our experimental results, even in the absence of any fit parameters. The simulations also reproduce the asymmetry of lower diffusivities toward the bromide side. This asymmetry can be understood by thinking about the dilute case. Exchanging some iodide with bromide ions in a (PEA)_2_PbI_4_ crystal (*x* = 0%) will result in a flat energy landscape with an occasional elevation in the potential *V*(***r***) (see Figure 4a). Excitons encountering such an elevation will not be slowed down significantly, as they simply scatter away from the obstacle or move around it. On the other hand, introducing iodide into a (PEA)_2_PbBr_4_ (*x* = 100%) lattice will result in valleys in the energetic landscape, where excitons can get stuck for a certain amount of time (see Figure 4a). Consequently, the impact of halide mixing on the transport properties is stronger on the bromide-rich side. Additionally, the asymmetry is enhanced by the smaller exciton Bohr radius *a*_B_^Br^ < *a*_B_^I^, which makes excitons on the bromide-rich side more susceptible to the local fluctuations in composition *x*′. However, as shown in SI, Figure S16, the asymmetry is clearly present even when assuming a constant exciton Bohr radius *a*_B_^I^ = *a*_B_^Br^ = *a*_B_(*x*) = 0.8 nm.

Brownian dynamics simulations also allow the extraction of the transient red-shifts (see SI, Figure S19). As shown in Figure 4c, the simulations also agree well with our transient spectroscopy measurements from Figure 3, reproducing the observed asymmetry and showing a good quantitative agreement for low alloying concentrations. It is worth highlighting that experimentally we observe a larger energetic red-shift in the range of *x* = 50–90% than suggested by the Brownian dynamics simulations. This could be due to uncertainties in our model, such as the exciton Bohr radius, which has a particularly strong impact on the energetic red-shift in this range or the approximation of the exciton probability density with a Gaussian function (see SI). Moreover, additional energetic disorder might be introduced by other alloying effects (e.g., strain), trap states, or self-trapped excitons, which are not included in our model. It is important to note that extending the model to account for a larger energy red-shift will also change the diffusivity, as the two properties are closely interrelated. However, a larger energetic red-shift will result in a lower diffusivity, hence both the simulated median emission energy and the simulated diffusivity should shift closer to our experimental observations. This is indeed what we observe for the simulation with smaller Bohr radii: *a*_B_^I^ = 1.05 nm and *a*_B_^Br^ = 0.6 nm, which corresponds to the lower edge of the shaded area in Figure 4b,c.

In conclusion, we have shown that halide mixing diminishes exciton transport, even in phase pure 2D perovskites, despite the absence of phase segregation. We find that the reduced diffusivity in 2D mixed-halide perovskites is a result of the intrinsic energetic disorder, caused by the *local* statistical fluctuations in halide composition, which restricts the movement of excitons. Our measurements show a significant reduction of exciton transport even at low levels of halide mixing. This effect is particularly strong on the bromide-rich side, where the diffusivity drops by more than an order of magnitude already for 5% or iodide alloying. Our experimental observations are supported by Brownian dynamics simulations, which successfully reproduce the spatial and energetic dynamics by only accounting for the presence of local statistical fluctuations in the halide composition. The good quantitative agreement between experiments and simulations is particularly impressive because no fitting parameters are used in our model. Our results highlight the importance of halide mixing on the spatial excited state dynamics in 2D metal-halide perovskites and should therefore be carefully considered in the design of optimized optoelectronic devices such as solar cells. For LEDs, however, exciton funneling and concentration at lower energy sites present an opportunity for improved LED performance as the increased local exciton concentrations allow radiative recombination to outperform trap-state mediated nonradiative recombination.^54^ On a final note, it is worth mentioning that most efforts to mitigate the negative impact of halide mixing in 3D perovskites have focused on the elimination of phase segregation.^12,32−34^ Crucially though, our results suggest that even if phase segregation is eliminated, halide mixing may still impact carrier transport due to the local intrinsic inhomogeneities in the energy landscape. While this study focuses exclusively on 2D perovskites, local statistical fluctuations in the halide composition will be present in any mixed-halide perovskite system and are therefore expected to impact carrier transport in other perovskite systems as well.

## Experimental Section

### Material Synthesis

2D perovskites of (PEA)_2_Pb(I_1–*x*_Br_*x*_)_4_ were grown following a simple supersaturation procedure under ambient conditions.^41,42^ First, two 0.2 M halide-pure stock solutions of (PEA)_2_PbX_4_ (X = I or Br) were prepared by mixing PEAX (Sigma-Aldrich: 805904-25G and 900829-10G) and PbX_2_ (Sigma-Aldrich: 900168-5G and 398853-5g) in a stoichiometric ratio (2:1) and dissolving the precursors in a 50/50 mixture of γ-butyrolactone and dimethyl sulfoxide (DMSO). The solutions were heated to 70 °C and stirred to accelerate the dissolution of the precursors. DMSO was needed to dissolve the PbBr_2_. Second, the I and Br stock solutions were mixed in a (1–*x*)/*x* ratio to obtain the final solutions of (PEA)_2_Pb(I_1–*x*_Br_*x*_)_4_. The (PEA)_2_Pb(I_1–*x*_Br_*x*_)_4_ solutions were dropcast onto a glass slide and after 1–3 days, millimeter-sized single-crystals formed, which were exfoliated using the scotch tape method and transferred to a coverslip for inspection.^43^

### Transient Photoluminescence Microscopy (TPLM)

TPLM measurements were performed following the procedure described by Akselrod et al.^45,46^ In short, a near-diffraction-limited exciton population was created using a 405 nm pulsed laser diode (PicoQuant LDH-D-C-405, PDL 800-D; 40 MHz, 50 nJ cm^–2^). The emission from the exciton population was optically magnified 330 times (Nikon CFI Plan Fluor, NA = 1.3) and imaged with a scanning avalanche photodiode (APD, Micro Photon Devices PDM, 20 × 20 μm^2^ detector). The laser diode and APD were synchronized by using a timing board for time-correlated single-photon counting (Pico-Harp 300). An *x*–*y* piezo stage (MCL Nano-BIOS 100) was used to scan the sample during the measurement, covering an area of 5 × 5 μm^2^, to reduce photodegradation of the perovskite flakes.

### Transient Photoluminescence Spectroscopy (TPLS)

TPLS measurements were performed with a Hamamatsu C10627 streak unit, which was coupled with a Hammamatsu C9300 digital camera and a SP2150i spectrograph (Princeton Instruments). Samples were excited with a 379 nm pulsed laser diode (Hammamatsu, 81 ps pulse width, 5 MHz, <5 nJ cm^–2^).

### Hyperspectral Imaging

Hyperspectral images of perovskite flakes were obtained with a SP2150i spectrograph (Princeton Instruments) and a piezo stage (MCL Nano-BIOS 100) using an oil immersion objective (NA = 1.3) and a 150× magnification. Perovskite flakes were excited with a 385 nm LED (Thorlabs M385PLP1-C5).

### Scanning Electron Microscopy (SEM) and Energy Dispersive X-ray Spectroscopy (EDS)

Perovskite crystals were mounted on a carbon tape on a stub, which were kept under vacuum for 15 min before loading into FESEM/EDS. Images were captured with a JEOL 6500F FESEM (15 kV) and an X-EDS spectrometer. The Oxford Instruments INCAEnergy+ software was used for elemental mapping.

### Brownian Dynamics Simulations

We modeled the motion of excitons as the diffusion of independent Brownian walkers satisfying the stochastic differential equation  in the standard Itô interpretation, where *F* = −∇*V* stands for the force felt by an exciton, *D*_0_ is the diffusion coefficient, *k*_B_*T* the thermal energy, and d*W* satisfies the Wiener process relation ⟨d*W*d*W*⟩ = Δ*t*. We integrated the equations numerically with the popular Euler–Maruyama scheme.

## Supporting Information Available

Additional material characterization, details of transient microscopy measurements and data analysis, and details of Brownian dynamics simulations. (PDF)
