# Supplementary material for: Halide Mixing Inhibits Exciton Transport in Two-dimensional Perovskites Despite Phase Purity
Source: ACS Energy Lett. 2021 Dec 22;7(1):358–65. doi: 10.1021/acsenergylett.1c02403 (PMC8762701; doi:10.1021/acsenergylett.1c02403)
Supplement: Supplementary file 1 — nz1c02403_si_001.pdf [file nz1c02403_si_001.pdf]

Supporting Information for

Halide Mixing Inhibits Exciton Transport in Two-Dimensional Perovskites Despite Phase Purity

*Michael Seitz[1,2,3,4], Marc Meléndez[5], Peyton York[6], Daniel A. Kurtz[3], Alvaro J. Magdaleno[1,2], Nerea Alcázar[1,5], Anuraj S. Kshirsagar[6], Mahesh K. Gangishetty[3,6], Rafael Delgado-Buscalioni[1,5], Daniel N. Congreve[3,4], and Ferry Prins[1,2]**

1. Condensed Matter Physics Center (IFIMAC), Autonomous University of Madrid, 28049 Madrid, Spain

2. Department of Condensed Matter Physics, Autonomous University of Madrid, 28049 Madrid, Spain

3. Rowland Institute at Harvard University, Cambridge, Massachusetts 02142, United States

4. Department of Electrical Engineering, Stanford University, Stanford, CA 94305, United States

5. Department of Theoretical Condensed Matter Physics, Autonomous University of Madrid, 28049 Madrid, Spain

6. Department of Chemistry, Mississippi State University, MS 39762, United States

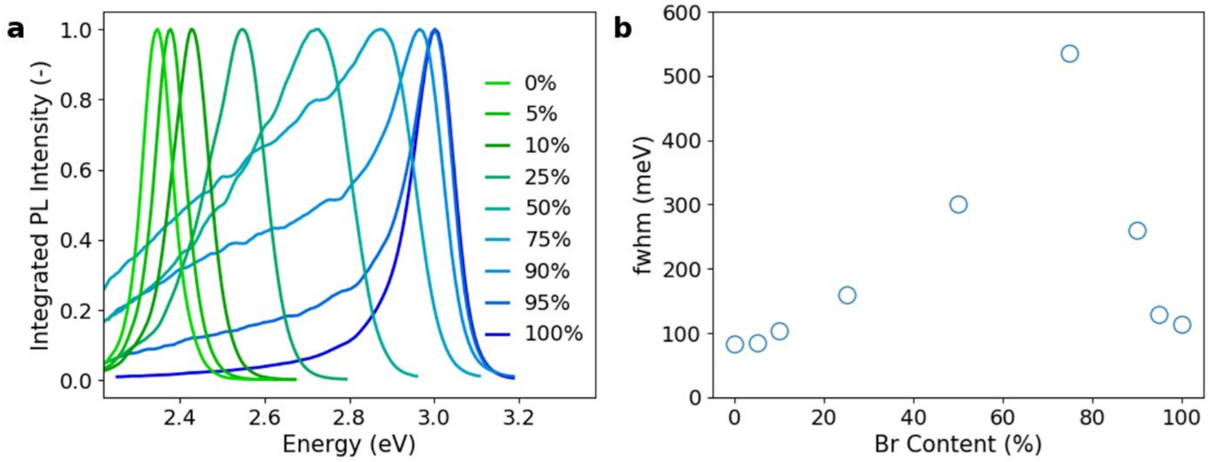

Figure S1. (a) Integrated photoluminescence (PL) emission from pulsed laser excitation of 2D metal halides $(PEA)_2Pb(I_{1-x}Br_x)_4$ with various bromide contents x = 0, 5, 10, 25, 50, 75, 90, 95, 100%. (b) Full-width-half-max (fwhm) of the PL spectra in a.

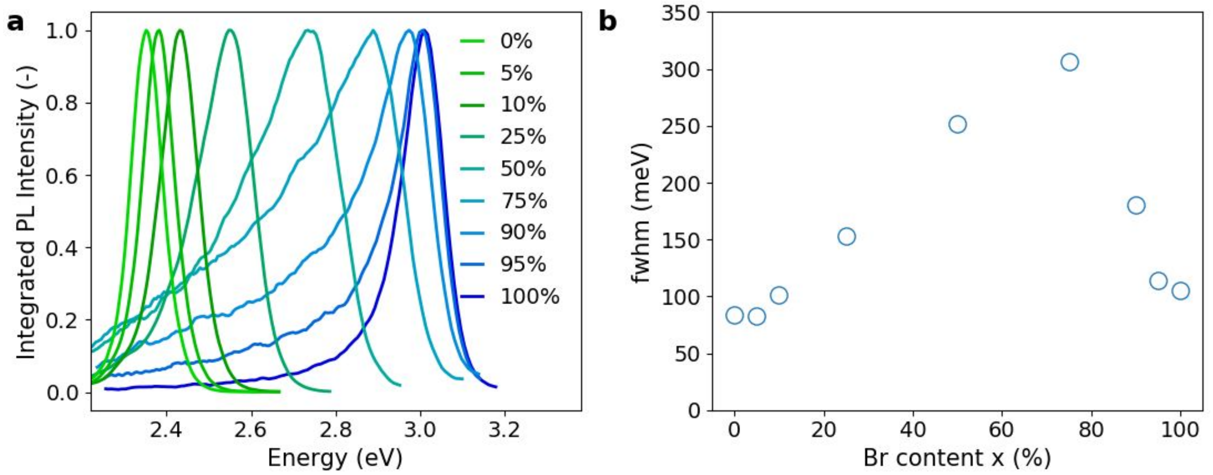

Figure S2. (a) Photoluminescence (PL) emission right after laser excitation (t = 0) of 2D metal halides $(PEA)_2Pb(I_{1-x}Br_x)_4$ with various bromide contents x = 0, 5, 10, 25, 50, 75, 90, 95, 100%. (b) Full-width-half-max (fwhm) of the PL spectra in a.

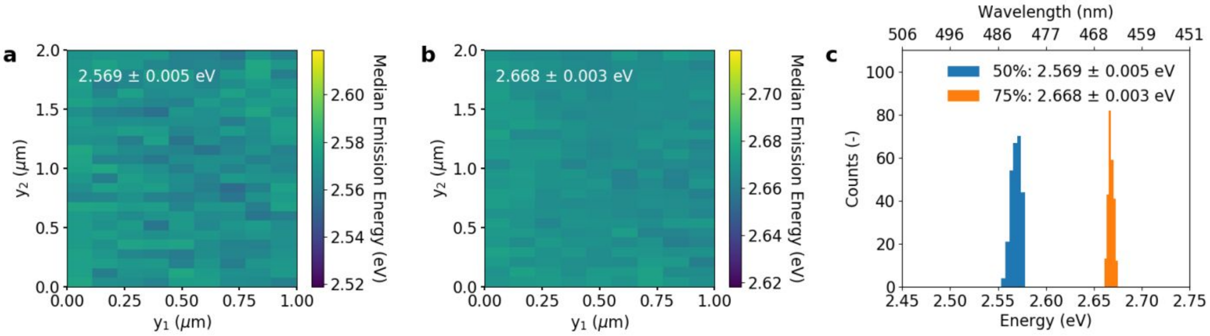

Figure S3. Hyperspectral imaging of $(PEA)_2Pb(I_{1-x}Br_x)_4$ crystals with x = 50 and 75%. (a, b) Median emission energy recorded of a 2 μm^2 area of crystals with x = 50% (a) and x = 75% (b). Scans were taken parallel to the inorganic crystal planes. Inset lists the average and standard deviation of the median emission energy in the recorded area. (c) Histogram of the median emission energy from a and b. Label shows the average and standard deviation of the median emission energy.

Energy Dispersive X-ray Spectroscopy (EDS). We performed EDS on a $(PEA)_2Pb(I_{0.5}Br_{0.5})_4$ (x = 50%) perovskite flake. The scan area is shown in Fig. S4 (left panel) as well as the measured iodide, bromide, and lead distributions. We find a homogeneous distribution of all atoms with no sign of phase segregation consistent with the hyperspectral images in Fig. S3. Further, the EDS scan confirms the x = 50% composition of the flake (see Table S1).

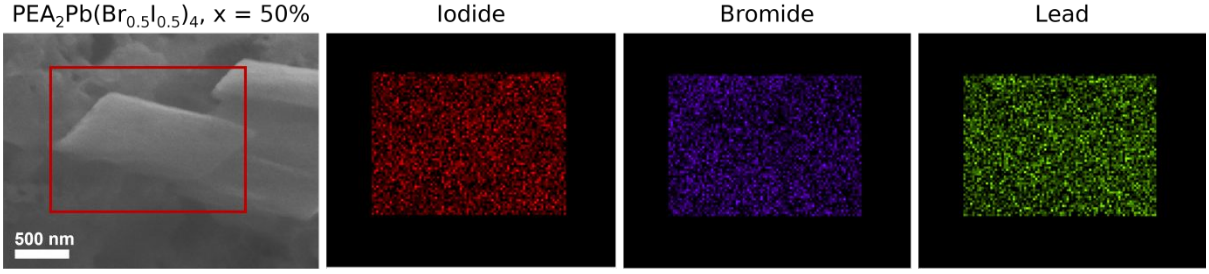

Figure S4. Energy dispersive X-ray spectroscopy (EDS) of a $(PEA)_2Pb(I_{0.5}Br_{0.5})_4$ (x = 50%) single crystal. Left image shows a scanning electron microscopy image of the crystal with the scan area highlighted with the red box. Images to the right show the iodide, bromide, and lead distribution obtained from the EDS scan.

Table S1: Elemental composition obtained with energy dispersive X-ray spectroscopy (EDS) of a $(PEA)_2Pb(I_{0.5}Br_{0.5})_4$ (x = 50%) perovskite flake (see also Fig. S4) confirming a bromide composition of 50%.

Element	Weight (%)	Atomic (%)
I	36.89	37.55
**Br**	23.25	37.59
**Pb**	39.86	24.85
**Total**		100

Transient photoluminescence microscopy (TPLM). TPLM was performed as previously reported.[1] In short, a near-diffraction limited exciton distribution was created using a ×100 oil immersion objective (Nikon CFI Plan Fluor, NA = 1.3) and a 405 nm pulsed laser diode (PicoQuant LDH-D-C-405, PDL 800-D). The photoluminescence of the exciton population was separated from the excitation light with a 420 nm dichroic mirror and imaged onto an avalanche photodiode (APD, Micro Photon Devices PDM, 20 µm detector size) with a ×330 magnification. APD and laser were synchronized using a timing board for time-correlated single photon counting (Pico-Harp 300). During TPLM the laser was scanned over a 5 × 5 µm area of the single-crystal (Mad City Labs Nano BIOS 100 x-y-piezo stage) to minimize photodegradation. The laser repetition rate was 40 MHz and the laser fluence was 50 $nJcm^{-2}$ unless stated otherwise. The time binning of the measurement setup was set to 4 ps before software binning was applied. The resulting diffusion maps are shown in Fig. S5. We follow the same fitting procedure as described previously by our group to extract the evolution of the mean-square-displacement (MSD) of the exciton population.[1] As shown in Fig. S6-8, the MSD first grows linearly and then transitions to a sublinear growth ($t > 1$ ns) due to excitons getting stuck at trapping sites.[1] We extract the diffusivity $D(x)$ of the different alloys using the one-dimensional diffusion equation and fitting the data to the linear regime at early times with $MSD(t) = 2D(x)t$ (see fit results in Fig. 2 of the main text).[1] The sensitivity of TPLM to exciton motion is limited by the signal-to-noise ratio of the experiment, rather than the diffraction limit. For this study, a detection limit of around 0.003 cm^2/s ($\approx \frac{\Delta MSD(4ns)_{noise}}{2 \cdot 4ns}$) for

the diffusivity is obtained. We would like to note that the absorption depth of 2D perovskites at 405 nm is around 100 nm, resulting in our TPLM measurements probing around 60 inorganic layers.[2]

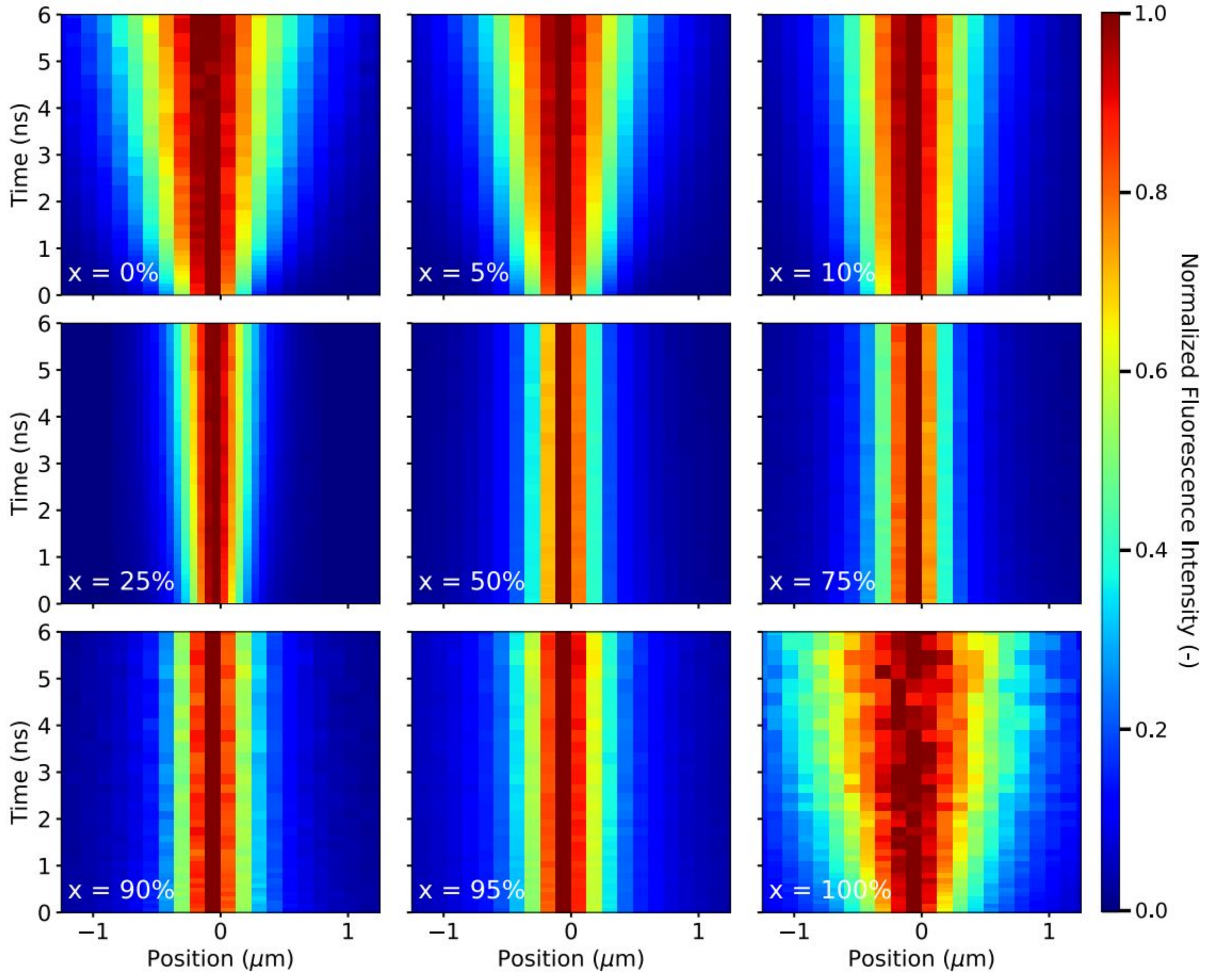

Figure S5. Diffusion maps for various mixed-halide perovskites $(PEA)_2Pb(I_{1-x}Br_x)_4$ with x = 0, 5, 10, 25, 50, 75, 90, 95, and 100%.

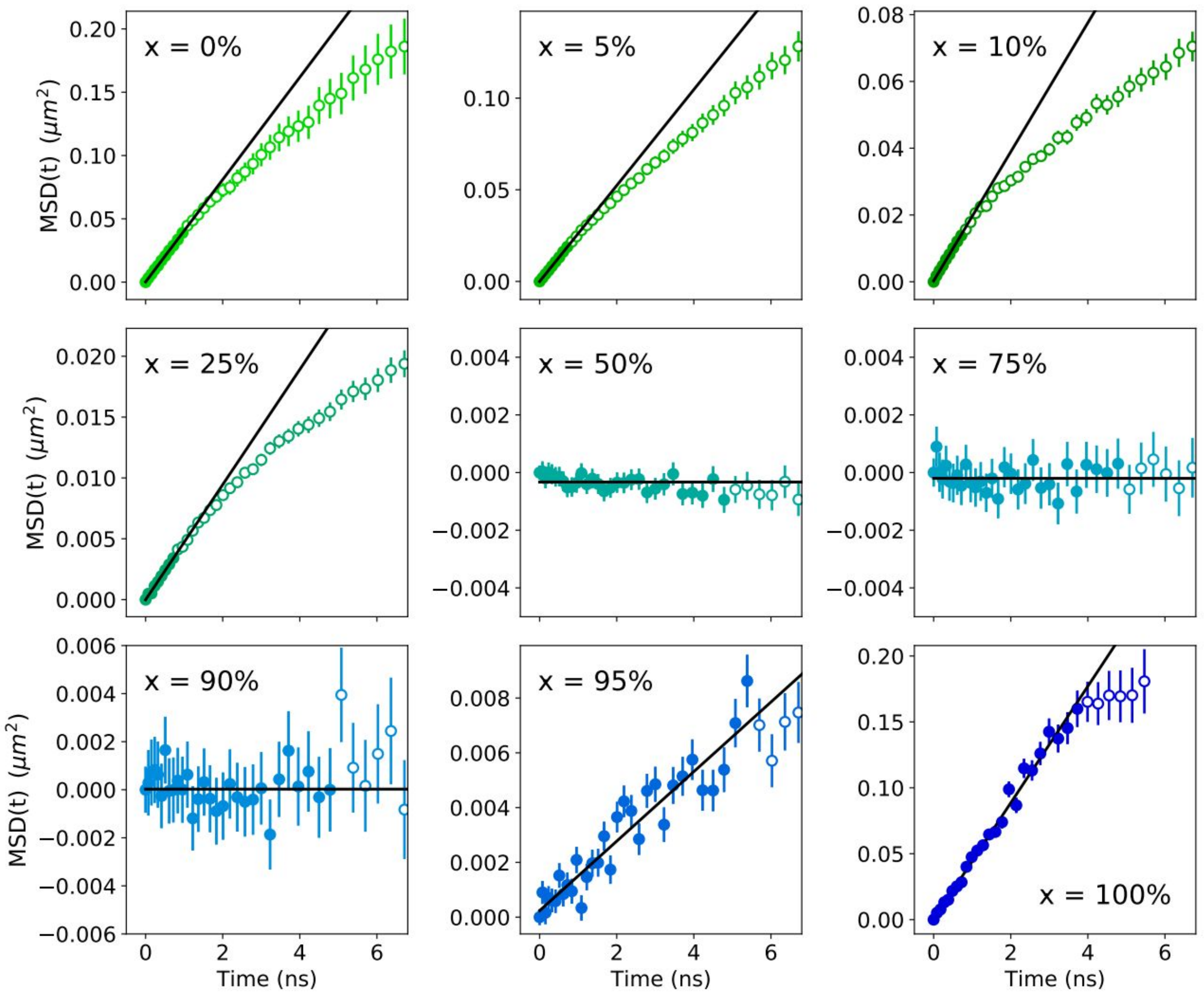

Figure S6. Evolution of the mean-square-displacement (MSD) of a near-diffraction limited exciton population in various mixed-halide perovskites $(PEA)_2Pb(I_{1-x}Br_x)_4$ with x = 0, 5, 10, 25, 50, 75, 90, 95, and 100%. Reported errors represent the uncertainty in the fitting procedure for $MSD(t)^2$.

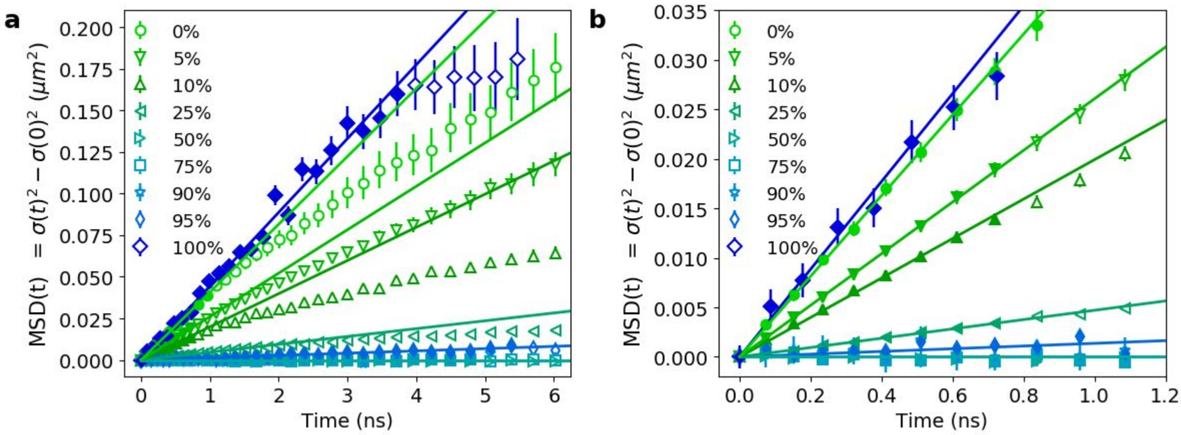

Figure S7. Evolution of the mean-square-displacement (MSD) of a near-diffraction limited exciton population in various mixed-halide perovskites $(PEA)_2Pb(I_{1-x}Br_x)_4$ with x = 0, 5, 10, 25, 50, 75, 90, 95, and 100%. (a) Up to 6 ns. (b) Up to 1 ns. Reported errors represent the uncertainty in the fitting procedure for $MSD(t)^2$.

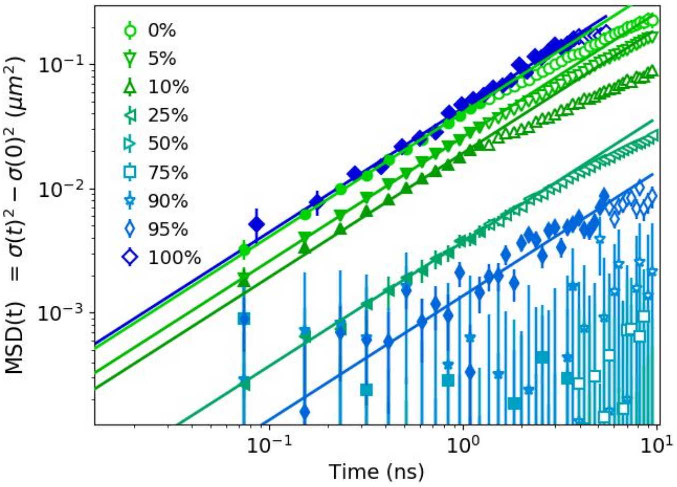

Figure S8. Log-log plot of the mean-square-displacement (MSD) of a near-diffraction limited exciton population in various mixed-halide perovskites $(PEA)_2Pb(I_{1-x}Br_x)_4$ with x = 0, 5, 10, 25, 50, 75, 90, 95, and 100%. Reported errors represent the uncertainty in the fitting procedure for $MSD(t)^2$.

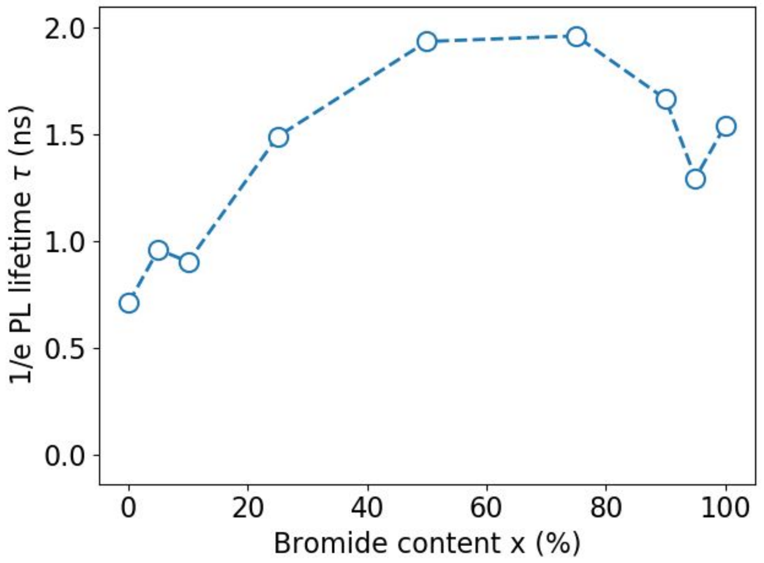

Figure S9. 1/e photoluminescence lifetime for various mixed-halide perovskites $(PEA)_2Pb(I_{1-x}Br_x)_4$ with x = 0, 5, 10, 25, 50, 75, 90, 95, and 100%.

For the x = 90, 95, and 100% alloys, part of the emission is blocked by the 420 nm dichroic mirror used in the optical setup. To check the impact of only using part of the emission spectrum for the TPLM measurements, we also performed TPLM with a 50/50 beam splitter instead of a 420 nm dichroic mirror on an x = 100% sample. The challenge with a 50/50 beam splitter is that light of the excitation laser also reaches the detector, which is why we had to attenuate the light reaching the APD, resulting in a reduced S/N of the measurement. To separate the laser light from the sample emission, we used the temporal resolution of TPLM since the laser signal decays faster than the photoluminescence of the perovskite. Looking at the photoluminescence decay measured with the 50/50 beam splitter (orange line in Fig. S10) we see that after around 0.6 ns (second dashed line in Fig. S10) the emission is dominated by photoluminescence from the perovskite (decay is comparable to photoluminescence measured with the dichroic mirror (blue line)). As a result, we only consider TPLM data after 0.6 ns for the evaluation of the MSD for the measurements with the 50/50 beam splitter (for measurements with the 420 nm dichroic we use data with t > 0 ns). The resulting MSD of the exciton population measured with a 50/50 beam splitter is shown in Fig. S11 (please note that t = 0 was shifted by 0.6 ns to have the MSD data start at 0 ns). The observed diffusivity of 0.245 cm^2s^{-1} is

comparable with our results obtained from measurements with a 420 nm dichroic mirror (0.222 cm^2s^{-1}, Fig. 1 of the main text). As a result, we conclude that the results from TPLM measurements with the 420 nm dichroic mirror represent the diffusivity of the x = 90, 95, 100% samples well, and we use the results from measurements with the dichroic mirror due to the superior S/N ratio of the data.

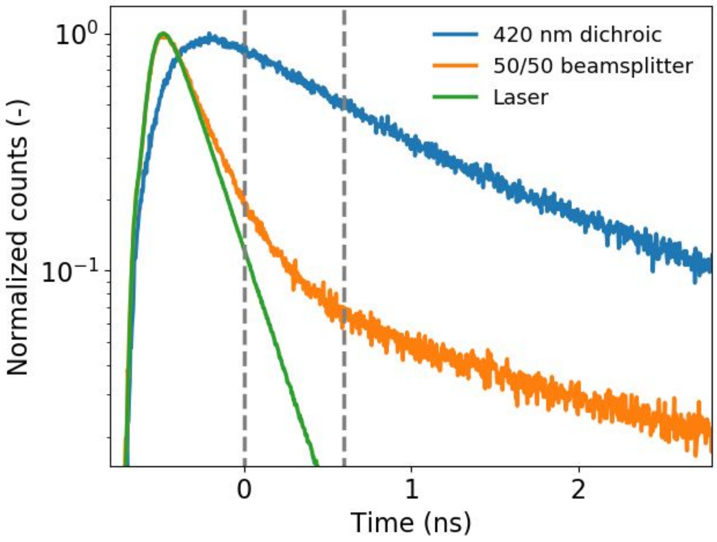

Figure S10. Photoluminescence lifetime traces of (PEA)PbBr4 (x = 100%) measured with a 420 nm dichroic mirror (blue line) and a 50/50 beam splitter (orange line). Green line shows the input response function of the excitation laser. With the dichroic mirror (blue line) the influence of the excitation is already negligible at 0 ns (first dashed line). With the 50/50 beam splitter (orange line) the laser excitation light is still significant until around 0.6 ns (second dashed line).

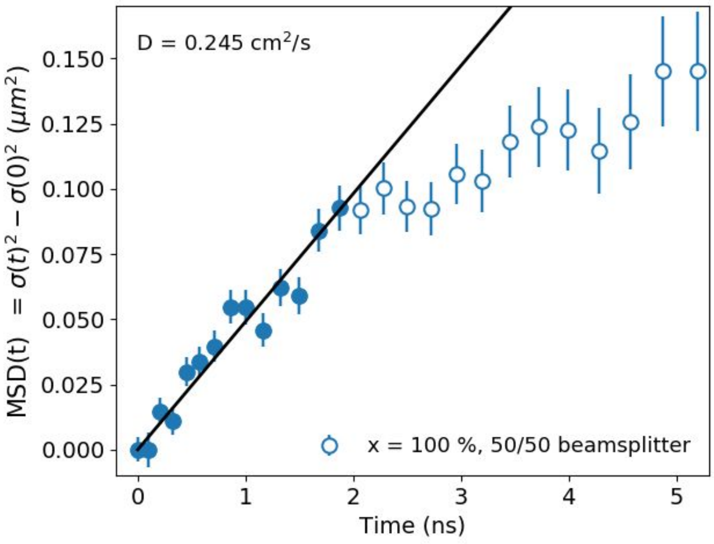

Figure S11. Evolution of the mean-square-displacement (MSD) of a near-diffraction limited exciton population in $(PEA)_2PbBr_4$ (x = 100%) measured with a 50/50 beam splitter. Please note that the time axis was shifted by 0.6 ns as compared to Fig. S10. Reported errors represent the uncertainty in the fitting procedure for $MSD(t)^2$.

Excitation fluence dependent TPLM. Perovskites are known to suffer from light induced phase segregation.[3–6] If light induced phase segregation is present in our measurements it should be stronger for higher laser fluences. Therefore, we performed TPLM measurements with two different excitation laser fluences: Our standard 50 nJcm^{-2} and a reduced 5 nJcm^{-2}. For this analysis, we focused on the x = 25% sample, which is the sample with the broadest PL emission peak still having a measurable diffusivity. In Fig. S12 we show that TPLM measurements with both laser fluences result in the same evolution of the MSD(t) and hence spatial dynamics. The MSD(t) for t > 1ns is slightly lower for 50 nJcm^{-2}. However, this is likely caused by the degradation of the perovskite flake (50 nJcm^{-2} was measured after the 5 nJcm^{-2} scan) as traps readily start affecting the later time dynamics.[7] As a result, we exclude photoinduced phase segregation to have a significant impact on the spatial dynamics observed in this study at the laser fluences that were used.

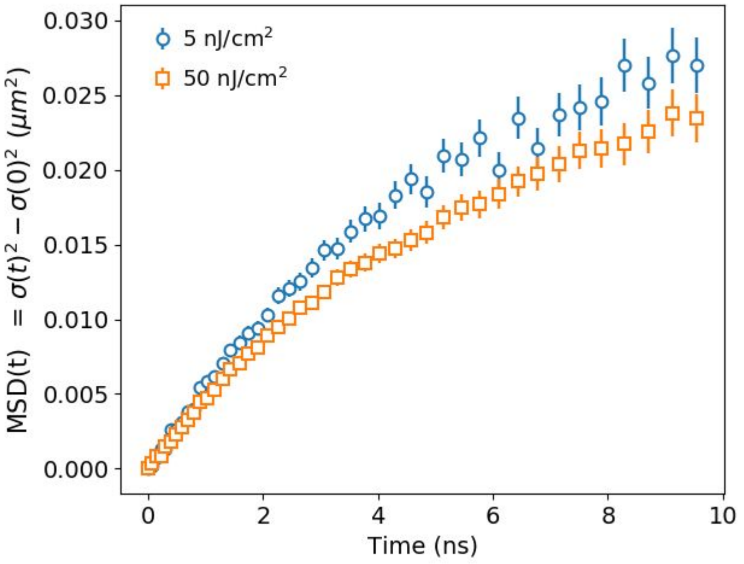

Figure S12. Evolution of the mean-square-displacement (MSD) of a near-diffraction limited exciton population in $(PEA)_2Pb(I_{0.75}Br_{0.25})_4$ (x = 25%) measured with two different excitation laser fluences: 5 nJcm^{-2} and 50 nJcm^{-2}. Reported errors represent the uncertainty in the fitting procedure for $MSD(t)^2$.

The fluence dependent TPLM measurements are also supported by fluence dependent spectral measurements. As shown in Fig. S13, the spectra do not change with increasing laser fluence which further demonstrates the stability of perovskite flakes at the fluences used in this study (50 nJ/cm^2).

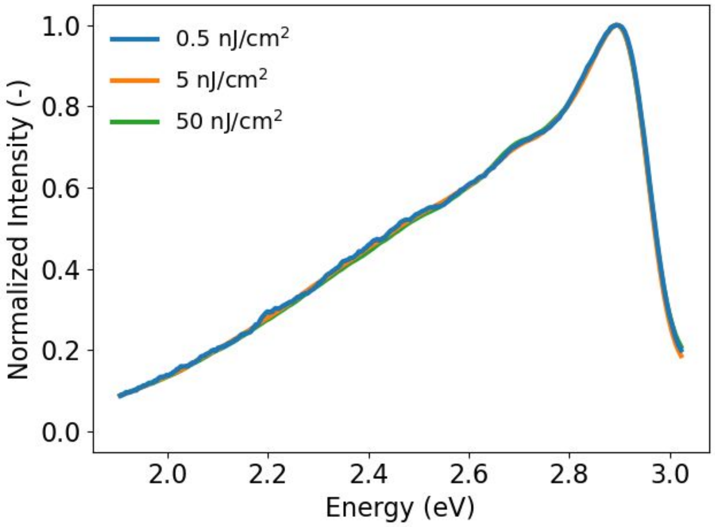

Figure S13. Fluence dependent emission spectra of a $(PEA)_2Pb(I_{1-x}Br_x)_4$ crystal with x = 75%. Before each spectra was taken the perovskite flake was exposed to the laser irradiation for 5 minutes.

Construction of V(r)

Crystal lattice. We extracted the lattice vectors and coordinates of iodide atoms in a $(PEA)_2PbI_4$ from reported single-crystal XRD data (see Table S2).[8] Please note that the out-of-plane coordinate was dropped to project the atoms on a 2D plane, defined by the lattice vectors **a** and **b**. In addition, all other atoms but halides (e.g. Pb) were neglected as they do not have an impact on the local halide composition experienced by the excitons. From $(PEA)_2PbI_4$[8] to $(PEA)_2PbBr_4$[9] the lattice contracts by a factor of (1 – 0.05845). As a result, we approximate the crystal lattices for all mixed-halide perovskites $(PEA)_2Pb(I_{1-x}Br_x)_4$ with the values listed in Table S2 for $(PEA)_2PbI_4$ and by scaling the lattice vectors with (1 – 0.05845·x), where x is the bromide content of the mixed-halide perovskite $(PEA)_2Pb(I_{1-x}Br_x)_4$.

Table S2: Lattice vectors and atom positions of iodide atoms in $(PEA)_2PbI_4$ as reported from single crystal XRD measurements.[8]

Lattice vectors (nm)	**a** = [0.87389, 0]<br>**b** = [0.00544589, 0.8740130]
Atom positions [y,z] in terms of the lattice vectors (y***a** + z***b**)	[0.691, 0.8084],<br>[0.4466, 0.4787],<br>[0.0164, 1.02],<br>[0.1905, 0.6906],<br>[-0.0164, 0.98],<br>[0.5534, 0.5213],<br>[0.309, 0.1916],<br>[0.8095, 0.3094]

Probability density of 2D excitons. In 2D systems, the interaction potential between electron and hole is best described by the Keldysh potential.[10,11] Solving the 2D Schrödinger equation yields a solution of the exciton wavefunction $\Psi(R)$, which lies between the 2D hydrogen model ($\Psi(R) \propto e^{-R/a_B}$) and a Gaussian function ($\Psi(R) \propto e^{-R^2/a_B^2}$), where a_B is the exciton Bohr radius and R is the distance from the center of the exciton.[10] For the main text, we decided to use the Gaussian approximation of the exciton with $\Psi(R) \propto e^{-R^2/a_B^2}$ and $|\Psi(R)|^2 \propto e^{-2R^2/a_B^2}$. Fig. S14 shows the results of Brownian dynamics simulations for both the Gaussian and the hydrogen model approximation. Simulations with the exact solution to the 2D Schrödinger equation with the Keldysh potential should lie between the two approximations.

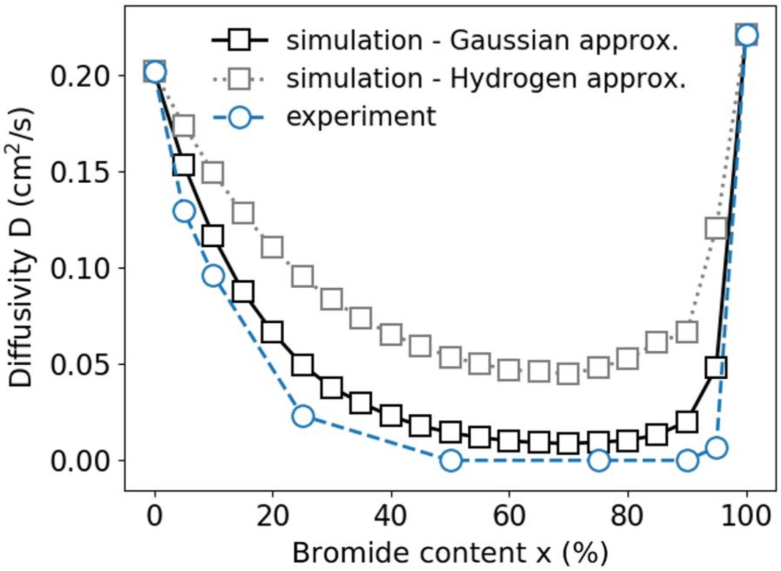

Figure S14. Diffusivities obtained from experiments (blue circles) and Brownian dynamics simulations using a Gaussian approximation (black squares) or the hydrogen approximation (gray squares) for the exciton wavefunction to generate the potential landscape $V(x'(\boldsymbol{r}))$. For both approximations, an exciton Bohr radius of $a_B(x) = (1-x)\cdot a_B^I + x\cdot a_B^{Br}$ with $a_B^I = 1.15\,nm$, $a_B^{Br} = 0.7\,nm$, and $r_{cutoff} = 3.5\,nm$ was used.

Local chemical halide composition x′. An exciton has a finite size that extends over several unit cells. In 2D, the exciton probability density can be approximated as $|\Psi(R)|^2 \propto e^{-2R^2/a_B^2}$, where a_B is the exciton Bohr radius and R is the distance from the center of the exciton. As a result, we approximate the local chemical composition x' observed by an exciton at position $\boldsymbol{r}$ as a weighted average of bromide and iodide atoms observed by the exciton: $x'(\boldsymbol{r}) = \frac{\sum_{|r_i - r| \le r_{cutoff}} X_i |\Psi(|\boldsymbol{r}_i - \boldsymbol{r}|)|^2}{\sum_{|r_i - r| \le r_{cutoff}} |\Psi(|\boldsymbol{r}_i - \boldsymbol{r}|)|^2}$, where $\boldsymbol{r}$ is the exciton position, $\boldsymbol{r_i}$ are the halide atom position, r_{cutoff} is the cutoff radius, X_i is a function that is 1 if the halide atom is bromide and 0 if the atom is iodide. r_{cutoff} was introduced to speed up calculations and was chosen to be more than five times larger than the standard deviation of the probability density $|\Psi(R)|^2$ (here $r_{cutoff} = 3.5\,nm > 5\frac{a_B(x)}{2} \forall a_B(x)$).

Potential landscape $\boldsymbol{\Delta V(x'(r))}$. For mixed-halide perovskites $(PEA)_2Pb(I_{1-x}Br_x)_4$, the optical bandgap changes linearly with the Bromide content x.[12] As a result, the bandgap observed by an exciton at position $\boldsymbol{r}$ can be calculated as: $E_g(x'(\boldsymbol{r})) = (1 - x') \cdot E_g^I + x' \cdot E_g^{Br}$, with $E_g^I = 2.400eV$ and $E_g^{Br} = 3.074eV$.[12] Brownian dynamics simulations were performed with these energetic landscapes (see Fig. S15). For the energy landscape, a constant offset can be introduced without impacting the dynamics. As a result, we use $V(\boldsymbol{r})$ ($= E_g(x'(\boldsymbol{r})) - \min\,[E_g(x'(\boldsymbol{r}))]$), where we subtract the minimal occurring bandgap energy, allowing a better visualization and comparison of the different potential landscapes $V(\boldsymbol{r})$.

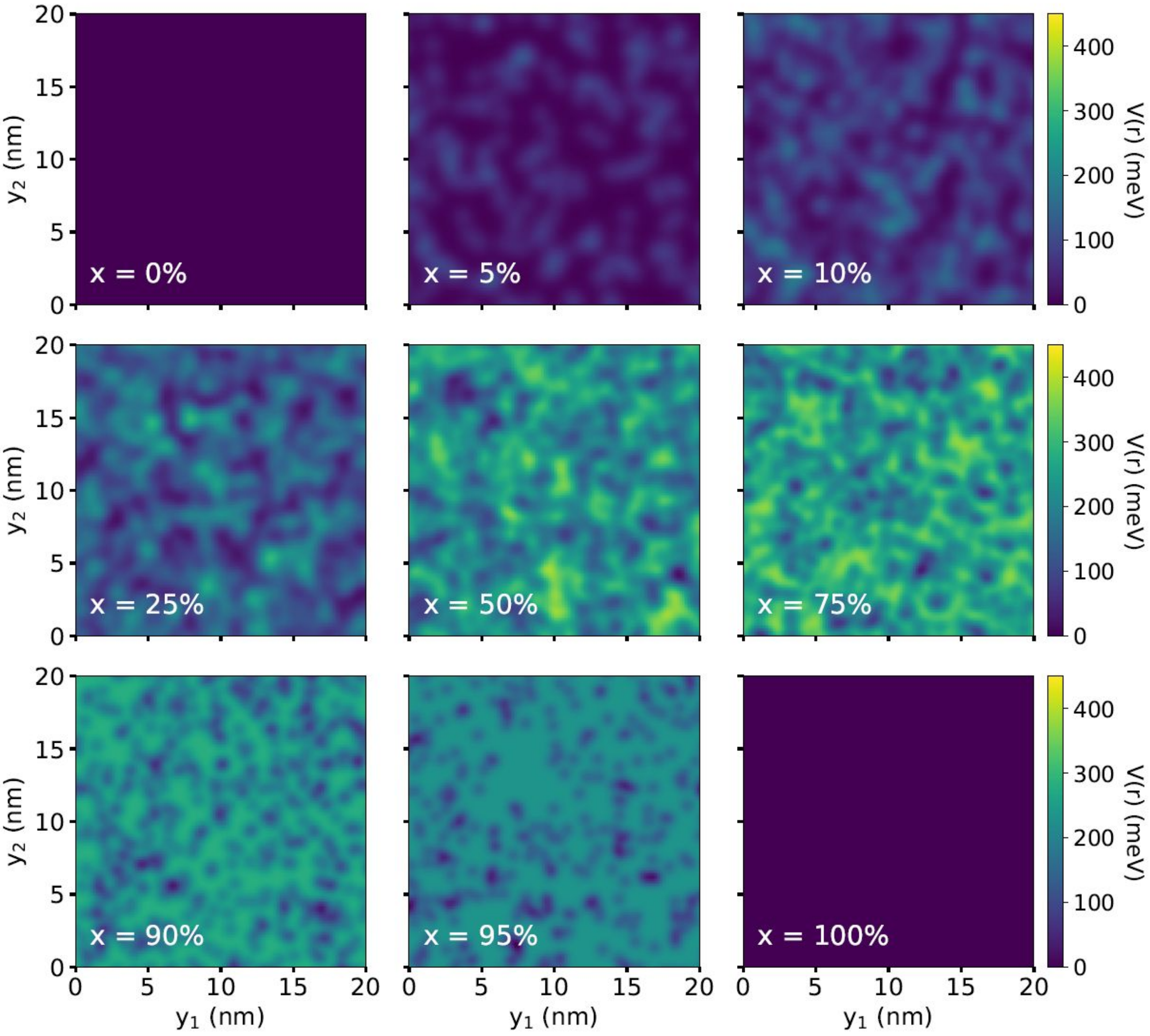

Figure S15. Potential landscapes $V(y_1,y_2)$ for different bromide contents x = 0, 5, 10, 25, 50, 75, 90, 95, and 100%.

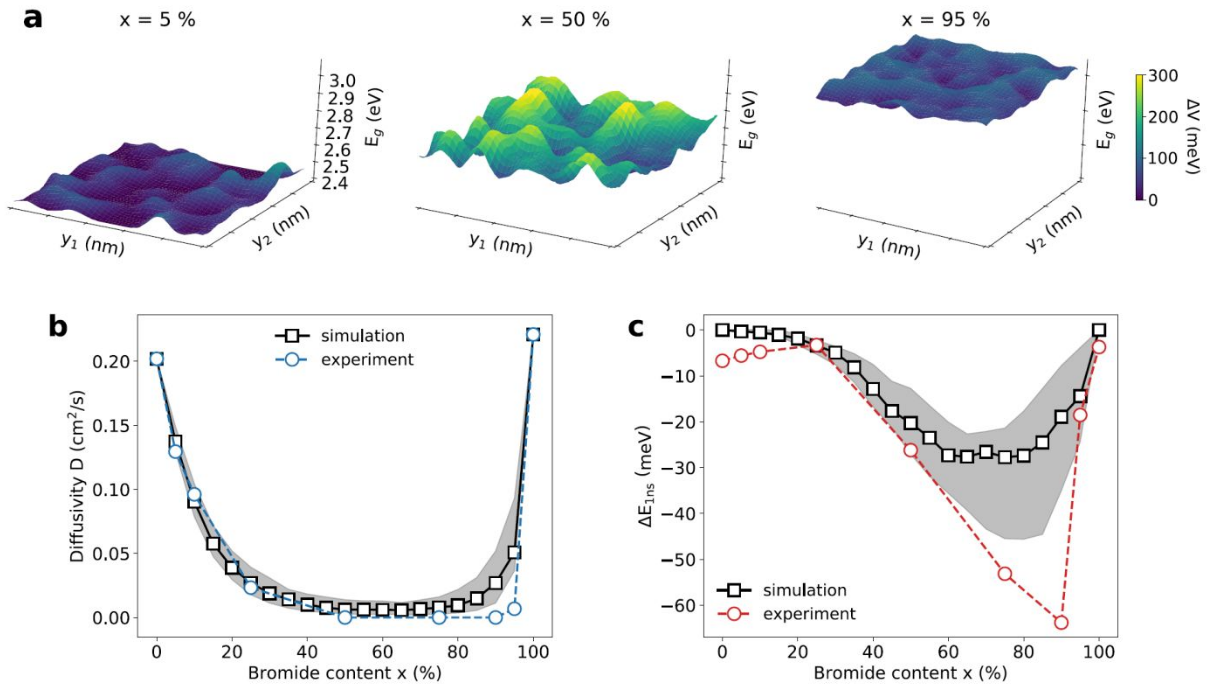

Figure S16. Same figure as Fig. 4 of the main text, but simulations were carried out with $a_B^I = a_B^{Br} = 0.8 \pm 0.1\, nm$. **a** Potential landscape for x = 5, 50, 95% for a 10 x 10 nm area. **b,c** Comparison of experiments (open circles) and Brownian dynamics simulations with an exciton Bohr radius of $0.8 \pm 0.1\, nm$ (open squares). It is worth noting that the asymmetry, with a steeper decay on the bromide rich side, is present despite assuming a constant exciton Bohr radius $a_B^I = a_B^{Br} = a_B(x) = 0.8 \pm 0.1\, nm$ for the simulations. We used a moving average ($f(x_i) = \frac{f(x_{i-1}) + f(x_i) + f(x_{i+1})}{3}$) for the simulated data points in **c** to reduce the noisiness. **b** Diffusivity $D(x)$ as a function of bromide content x. **c** Change in median energy after 1 ns of photoexcitation as a function of bromide content x.

Brownian dynamics simulations. We simulated the diffusion process as Brownian walkers in a potential landscape which modeled the interactions between excitons and the crystal structure. The trajectories were represented by the standard stochastic differential motion equations in the Itô interpretation: $\Delta \boldsymbol{r} = \frac{D}{k_B T}\boldsymbol{F}\Delta t + \sqrt{2D_0}d\boldsymbol{W}$, where $\boldsymbol{F} = -\nabla V$ stands for the force felt by an exciton, D_0 is the diffusion coefficient, $k_B T$ is the thermal energy and $d\boldsymbol{W}$ is taken from a Wiener process, such that $\langle d\boldsymbol{W} d\boldsymbol{W} \rangle = \Delta t$. Numerical integration was carried out with the straightforward Euler-Maruyama method.

Excitons moved in a two-dimensional 250 x 250 unit cell rectangular simulation box with periodic boundary conditions. As a consequence, the boundaries introduced periodic line crystal defects in the x direction separated by 250 cells in the y direction. We believe these defects had a negligible effect on the simulation results.

At time $t = 0$, excitons were placed radially following a Gaussian distribution of standard deviation $\sigma_0 = 25\ nm$, and the change in the distribution variance was tracked for 2 ns. The program also recorded the evolution of the exciton energy distribution from which we could later calculate the interpolated medians. For each value of the bromide content x, we ran the simulation four times, always generating a new potential landscape for each run and averaging the final results. The one-dimensional MSD was calculated with $MSD(t) = \frac{1}{2}(MSD_x(t) + MSD_y(t))$.

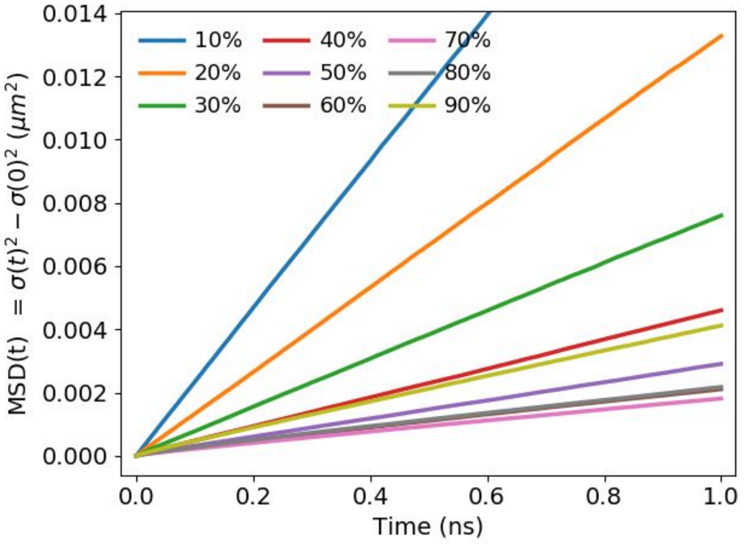

Figure S17. Mean-square-displacement (MSD) of the simulations with x = 10, 20, 30, 40, 50, 60, 70, 80, and 90% as shown in Fig. 4b in the main text.

We would like to highlight that the diffusivities $D(x)$ shown in Fig. 4b, Fig. S16b, and represented by the slope of the MSD in Fig. S17 are effective diffusivities, which are lower than the intrinsic diffusion coefficients $D_0(x)$ that one would observe for a flat energy landscape: $D(x) < D_0(x)$. This is a result of the inhomogeneous energy landscape which restricts the movement of excitons as

they get stuck at low-energy sites or move along percolation paths. At very early times, when excitons have not traveled far enough to experience the inhomogeneity of the potential landscape, excitons travel with a diffusivity close to $D_0(x)$.[7,13] However, after less than one picosecond, excitons start experiencing the local disorder resulting in the reduced effective diffusivity $D(x)$ as is shown in Fig. S18. The time scale at which this transition happens coincides with the time needed to travel a few nanometers – the characteristic length scale of the inhomogeneous energy landscape ($4\,nm^2/D_0/2 \approx 100\,fs$, see Fig. S18). While we are able to resolve these timescales with our simulations, they are, unfortunately, not accessible with our experimental setup. Consequently, we only used $D(x)$ to compare experiments and simulations.

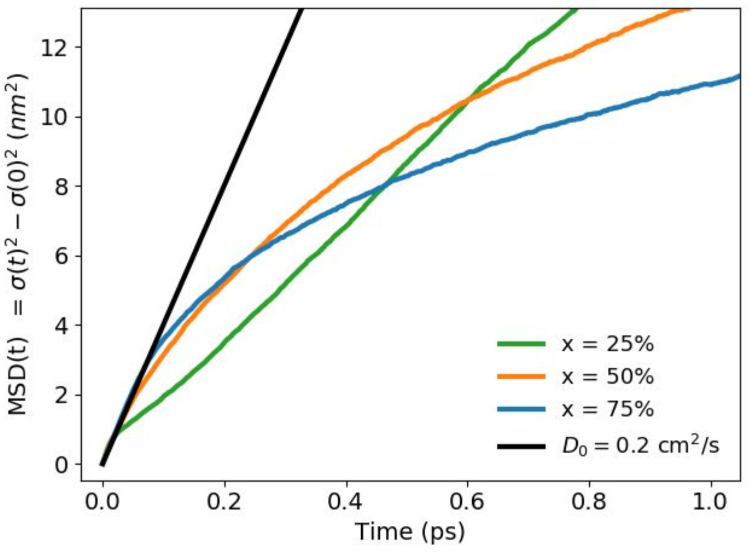

Figure S18. Mean-square-displacement (MSD) of the simulations for x = 25, 50, and 75% for early time ($\lesssim$ 1ps). Showing the transition of a broadening proportional to the diffusion coefficient D0 to a slower effective diffusivity D(x) due to the energetic disorder.

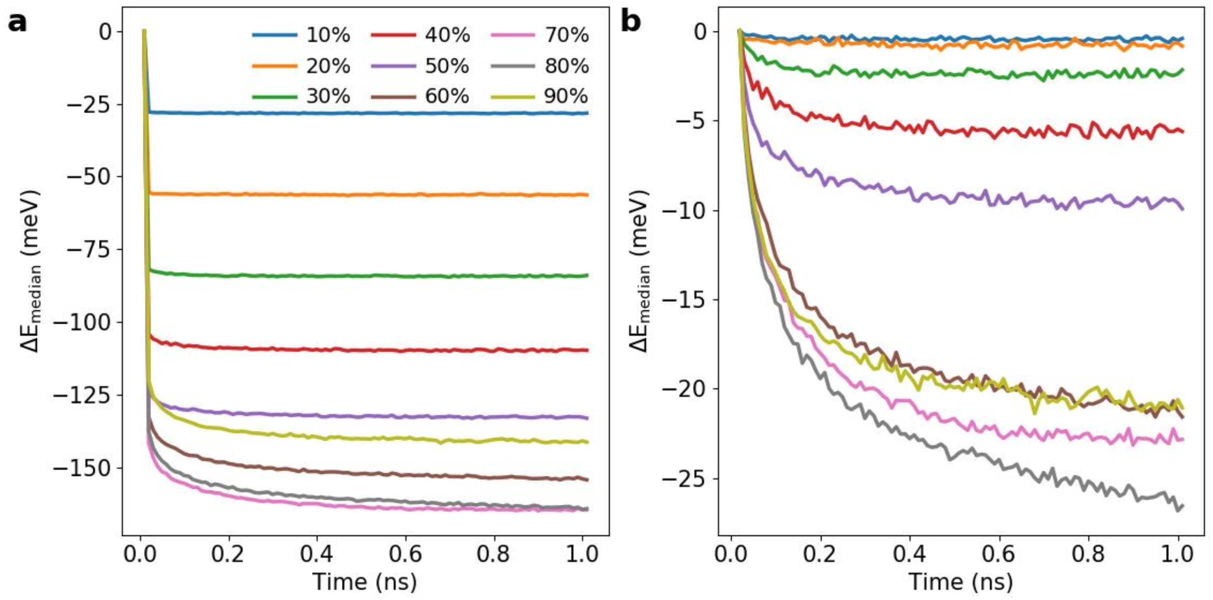

Figure S19. a Change of the median energy of the exciton populations shown in Fig. 4c. **b** Same as **a** but without the energy shift within the first 10 ps. Value of $\Delta E_{median}(1\ ns)$ from **b** was taken for the comparison for the experimental data in Fig. 4c.

Supplementary References

(1) Seitz, M.; Magdaleno, A. J.; Alcázar-Cano, N.; Meléndez, M.; Lubbers, T. J.; Walraven, S. W.; Pakdel, S.; Prada, E.; Delgado-Buscalioni, R.; Prins, F. Exciton Diffusion in Two-Dimensional Metal-Halide Perovskites. *Nat. Commun.* **2020**, *11* (1), 2035. https://doi.org/10.1038/s41467-020-15882-w.

(2) Zhang, S.; Audebert, P.; Wei, Y.; Choueiry, A. Al; Lanty, G.; Bréhier, A.; Galmiche, L.; Clavier, G.; Boissiére, C.; Lauret, J. S.; et al. Preparations and Characterizations of Luminescent Two Dimensional Organic-Inorganic Perovskite Semiconductors. *Materials* **2010**, *3* (5), 3385. https://doi.org/10.3390/MA3053385.

(3) Hoke, E. T.; Slotcavage, D. J.; Dohner, E. R.; Bowring, A. R.; Karunadasa, H. I.; McGehee, M. D. Reversible Photo-Induced Trap Formation in Mixed-Halide Hybrid Perovskites for Photovoltaics. *Chem. Sci.* **2015**, *6* (1), 613–617. https://doi.org/10.1039/c4sc03141e.

(4) Bischak, C. G.; Hetherington, C. L.; Wu, H.; Aloni, S.; Ogletree, D. F.; Limmer, D. T.; Ginsberg, N. S. Origin of Reversible Photoinduced Phase Separation in Hybrid Perovskites. *Nano Lett.* **2017**, *17* (2), 1028–1033. https://doi.org/10.1021/acs.nanolett.6b04453.

(5) Brennan, M. C.; Draguta, S.; Kamat, P. V.; Kuno, M. Light-Induced Anion Phase Segregation in Mixed Halide Perovskites. *ACS Energy Lett.* **2018**, *3* (1), 204–213. https://doi.org/10.1021/acsenergylett.7b01151.

(6) Knight, A. J.; Wright, A. D.; Patel, J. B.; McMeekin, D. P.; Snaith, H. J.; Johnston, M. B.; Herz, L. M. Electronic Traps and Phase Segregation in Lead Mixed-Halide Perovskite. *ACS Energy Lett.* **2019**, *4* (1), 75–84. https://doi.org/10.1021/acsenergylett.8b02002.

(7) Seitz, M.; Meléndez, M.; Alcázar-Cano, N.; Congreve, D. N.; Delgado-Buscalioni, R.; Prins, F. Mapping the Trap-State Landscape in 2D Metal-Halide Perovskites Using Transient Photoluminescence Microscopy. *Adv. Opt. Mater.* **2021**, 2001875. https://doi.org/10.1002/adom.202001875.

(8) Du, K. Z.; Tu, Q.; Zhang, X.; Han, Q.; Liu, J.; Zauscher, S.; Mitzi, D. B. Two-Dimensional Lead(II) Halide-Based Hybrid Perovskites Templated by Acene Alkylamines: Crystal Structures, Optical Properties, and Piezoelectricity. *Inorg. Chem.* **2017**, *56* (15), 9291–9302. https://doi.org/10.1021/acs.inorgchem.7b01094.

(9) Gong, X.; Voznyy, O.; Jain, A.; Liu, W.; Sabatini, R.; Piontkowski, Z.; Walters, G.; Bappi, G.; Nokhrin, S.; Bushuyev, O.; et al. Electron-Phonon Interaction in Efficient Perovskite Blue Emitters. *Nat. Mater.* **2018**, *17* (6), 550–556. https://doi.org/10.1038/s41563-018-0081-x.

(10) Prada, E.; Alvarez, J. V.; Narasimha-Acharya, K. L.; Bailen, F. J.; Palacios, J. J. Effective-Mass Theory for the Anisotropic Exciton in Two-Dimensional Crystals: Application to Phosphorene. *Phys. Rev. B* **2015**, *91* (24), 245421. https://doi.org/10.1103/PhysRevB.91.245421.

(11) Keldysh, L. V. Coulomb Interaction in Thin Semiconductor and Semimetal Films. *J. Exp. Theor. Phys. Lett.* **1979**, *29*, 658.

(12) Lanty, G.; Jemli, K.; Wei, Y.; Leymarie, J.; Even, J.; Lauret, J. S.; Deleporte, E. Room-Temperature Optical Tunability and Inhomogeneous Broadening in 2D-Layered Organic-Inorganic Perovskite Pseudobinary Alloys. *J. Phys. Chem. Lett.* **2014**, *5* (22), 3958–3963. https://doi.org/10.1021/jz502086e.

(13) Delor, M.; Slavney, A. H.; Wolf, N. R.; Filip, M. R.; Neaton, J. B.; Karunadasa, H. I.; Ginsberg, N. S. Carrier Diffusion Lengths Exceeding 1 Mm despite TraLimited Transport in Halide Double Perovskites. *ACS Energy Lett.* **2020**, 1337–1345. https://doi.org/10.1021/acsenergylett.0c00414.
